# Syndromic approaches for sexually transmitted infections: added value of molecular diagnosis

**DOI:** 10.1097/COH.0000000000000932

**Published:** 2025-04-01

**Authors:** Onya Opota, Gilbert Greub

**Affiliations:** a Institute of Microbiology; b Infectious Disease Service, University of Lausanne and Lausanne University Hospital, Lausanne (Vaud), Switzerland

**Keywords:** PCR, sexually transmitted infection, syndromic diagnostics

## Abstract

**Purpose of review:**

Sexually transmitted infections (STIs) are a significant global health concern, with many cases going undiagnosed due to asymptomatic infections. Traditional diagnostic methods, such as culture and serology, have limitations in sensitivity, specificity, and turnaround time. Molecular diagnostics, particularly PCR-based approaches, offer significant advantages, including improved detection rates and the potential for syndromic testing. This review examines the role of syndromic PCR diagnostics in improving STI detection and management.

**Recent findings:**

Recent studies highlight the utility in detecting common STIs, such as *Chlamydia trachomatis*, *Neisseria gonorrhoeae*, and *Trichomonas vaginalis*, as well as emerging pathogens. PCR-based syndromic panels allow for the simultaneous detection of multiple pathogens from a single sample, improving diagnostic accuracy and efficiency. Syndromic PCR approaches streamline diagnosis, aid in early detection, and support efficient treatment, addressing both common and emerging infections.

**Summary:**

Syndromic PCR diagnostics streamline STI detection, addressing the limitations of conventional methods. They enable faster, more accurate, and comprehensive diagnosis, leading to targeted treatment and improved patient outcomes. Expanding syndromic panels to include emerging pathogens and ensuring cost-effective implementation remain key areas for future research.

## INTRODUCTION

Sexually transmitted infections (STIs) remain a major global public health challenge, affecting millions of persons annually and causing significant morbidity and reproductive health issues [1]. They manifest in various conditions, such as urethritis, proctitis, pharyngitis, vaginitis, cervicitis, and pelvic inflammatory disease (PID), but can also be asymptomatic, which may lead to underestimating their true prevalence (Table 1) [2^▪^]. Moreover, vertical transmission during pregnancy, childbirth, or breastfeeding poses significant risks [3]. STI can be caused by various bacteria, viruses, and parasites transmitted through sexual contact, including well known pathogens such as *Chlamydia trachomatis*, *Neisseria gonorrhoeae*, *Treponema pallidum* (syphilis), *Trichomonas vaginalis*, *HSV*, *HBV*, and *HIV*. Emerging infections like Monkeypox, *Shigella sonnei*, *Neisseria meningitidis*, and Zika virus, as well as a resurgence of neglected STIs like lymphogranuloma venereum, add complexity [4,5]. Despite advances in diagnostics, STI management faces challenges due to the asymptomatic nature of many infections and the overlapping clinical presentation with non-specific symptoms. Co-infections and the growing threat of antimicrobial resistance (AMR) further complicate treatment. Some STIs, such as herpes, gonorrhea, syphilis, and HPV, increase susceptibility to other infections, including HIV [6^▪▪^]. This review focuses on non-HIV STIs, highlighting current screening and diagnostic methods, with a particular emphasis on PCR-based syndromic approaches. These methods have revolutionized STI diagnostics by enabling the identification of multiple pathogens based on clinical syndromes. Syndromic testing supports timely empirical treatment, preventing further transmission and reducing disease burden. 

**Box 1:**
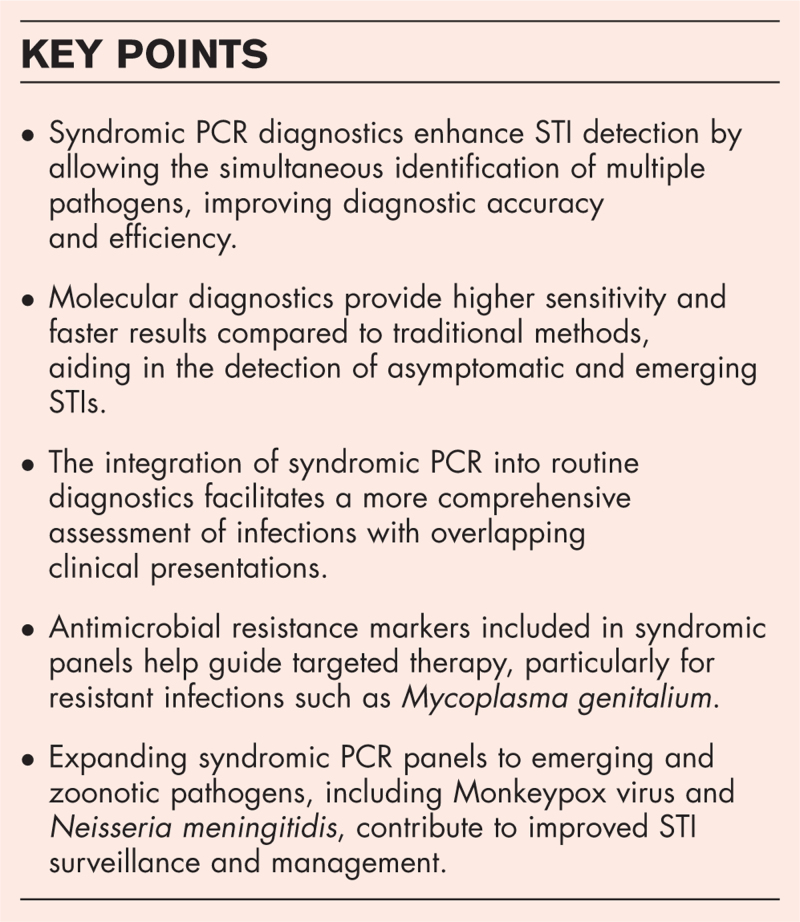
no caption available

**Table 1:** Advantages and limits of diagnostic methods for sexually transmitted infections

Diagnostic method	Advantages	Limits	Ideal use cases
Serology	Detect past infections Can provide rapid results	Time to development of the serological response Inability to distinguish between past and active infections	Detection of viral STIs after the development of the serological response (HIV, HBV, syphilis)
Microscopy	Quick assessment of sample	Low sensitivity (both for symptomatic and asymptomatic infections) Requires expertise	Quick assessment for bacterial vaginosis or candidiasis
Culture	Allows for antimicrobial susceptibility testing Effective for nonfastidious organisms	Time-consuming Inefficient for fastidious organisms	Antimicrobial susceptibility testing, especially for bacterial pathogens
PCR	High sensitivity and specificity Rapid turnaround Multiplexing capabilities Detects nonculturable pathogens Quantification capabilities Retesting preserved nucleic acids	Specialized infrastructure and equipment Cost Limited accessibility in resource-limited settings Contamination risk Does not provide live isolates for susceptibility testing	Syndromic diagnosis of STIs (e.g., simultaneous detection of *C. trachomatis, N. gonorrhoeae*) Retesting preserved nucleic acids Quantitative detection of pathogens

## ADVANTAGES AND LIMITATIONS OF MOLECULAR DIAGNOSTICS FOR SEXUALLY TRANSMITTED INFECTIONS

Molecular diagnostics, particularly PCR, have transformed the diagnosis of infectious diseases in general and STIs in particular, offering significant advantages over traditional methods. While microscopy-based methods (e.g., Nugent score) and culture-based techniques remain essential for certain applications, like bacterial vaginosis or candidiasis, using culture on selective media and are still in use, they are less practical for rapid and specific diagnosis. PCR provides high sensitivity and specificity, detecting intracellular and fastidious pathogens such as *C. trachomatis* and *N. gonorrhoeae* (Table 1). PCR includes various testing formats, including monoplex PCR, which targets a single pathogen, which is ideal for focused diagnostics, multiplex PCR, which detects two to three pathogens in one assay, useful for infections with overlapping symptoms, and syndromic panels high-plex systems capable of identifying up to 20 pathogens or more simultaneously, which offers comprehensive diagnosis. Technologies such as TaqMan, along with alternative platforms, enable precise pathogen quantification and rapid results delivery. For example, syndromic panels streamline diagnostics for urethral discharge by simultaneously testing for *C. trachomatis* and *N. gonorrhoeae*, expediting treatment decisions. PCR's flexibility allows for preserved nucleic acids in sample tubes to be retested if initial results are negative, minimizing the need for repeat sampling. Despite its advantages, PCR has limitations, such as the inability to provide live isolates for susceptibility testing or detect past infections, which remain the domain of serological methods mainly used for STIs like HIV, HBV, and syphilis (Table 1).

## PCR-BASED SYNDROMIC APPROACH FOR PATHOGEN DETECTION

### Urogenital infections

Urethritis is commonly caused by *C. trachomatis* and *N. gonorrhoeae*. *C. trachomatis* often presents asymptomatically, while *N. gonorrhoeae* generally cause symptomatic infections [7]. PCR-based assays detect bacterial DNA from noninvasive samples like urine or swabs, offering superior sensitivity and rapid results compared to culture, which is time-consuming and limited by the fastidious nature of *N. gonorrhoeae*[8][8–10]. PCR allows for simultaneous detection of both pathogens in a single assay, streamlining diagnosis and treatment (Table 2). Many assays also target additional pathogens, such as *Mycoplasma genitalium*, *M. hominis*, and *Ureaplasma urealyticum*, expanding diagnostic capability for nongonococcal urethritis (NGU) cases [4,11]. *M. genitalium* is a significant cause of NGU and cervicitis, often resistant to common antibiotics, making targeted PCR testing crucial. Stepwise PCR testing is indicated for *M. hominis*, and *U. urealyticum* via quantitative or semi-quantitative PCR after excluding *M. genitalium*[12]. PCR offers the advantage of directly identifying this pathogen from urine or swabs, enabling targeted treatment, particularly since *M. genitalium* is often resistant to commonly used antibiotics. *U. urealyticum* is also often associated with nongonococcal urethritis. The screening of asymptomatic individual is not recommended; however, detection of these pathogens is indicated for NGU negative for *M. genitalium*[12]. For pregnant women, targeted PCR for *M. hominis* and *U. urealyticum* is advised in symptomatic cases, such as preterm labor or rupture of membranes, after excluding *N. gonorrhoeae*, *C. trachomatis*, and *M. genitalium*. This species-specific approach minimizes unnecessary broad testing while improving clinical impact of testing. Similarly, PCR aids in diagnosing orchitis and epididymitis, often caused by *C. trachomatis* or *N. gonorrhoeae*, particularly in sexually active males (Table 2). Cervicitis involves similar pathogens, with *M. genitalium* being increasingly implicated. PCR's ability to detect low-level or mixed infections helps prevent complications like PID, a severe consequence of *C. trachomatis*, *N. gonorrhoeae*, or *M. genitalium* infections [13^▪▪^]. Syndromic PCR allows for early PID diagnosis and co-infection detection, reducing risks such as infertility and chronic pelvic pain thanks to early appropriate treatment [14]. This approach is also beneficial for diagnosing salpingitis and bartholinitis, preventing severe reproductive complications due to ongoing infections. For the diagnosis of vaginitis caused by *Trichomonas vaginalis,* PCR is particularly valuable for asymptomatic cases or low parasite loads ensuring accurate detection and facilitating the diagnosis of mixed infections. Recent studies supported the use of PCR for accelerated diagnosis of candidiasis [15–17]. For genital ulcers, the use of PCR significantly improves the detection of common pathogens such as *Treponema pallidum* (syphilis), *Herpes simplex virus* (HSV), and less frequently *Haemophilus ducreyi* or *Klebsiella granulomatis*[18]. PCR complements serology in early stage of the disease, where serological tests may be unreliable. PCR is also the preferred method for detecting HSV-1 and HSV-2 due to its rapid and specific results (Table 2). Condylomata (genital warts) are linked to human papillomavirus (HPV), the most common viral STI. High-risk HPV strains, including HPV-16 and HPV-18, significantly increase the risk of cervical and other anogenital cancers and the risk of other STI such as HIV [6^▪▪^,^19^]. PCR-based HPV assays detect viral DNA and identify strains associated with high-risk of HPV, playing a critical role in screening and monitoring persistent infections. This ensures timely interventions, reducing cancer risk and complications such as infertility and incontinence. Emerging evidence highlights the need for assays covering all high-risk HPV strains.

**Table 2:**
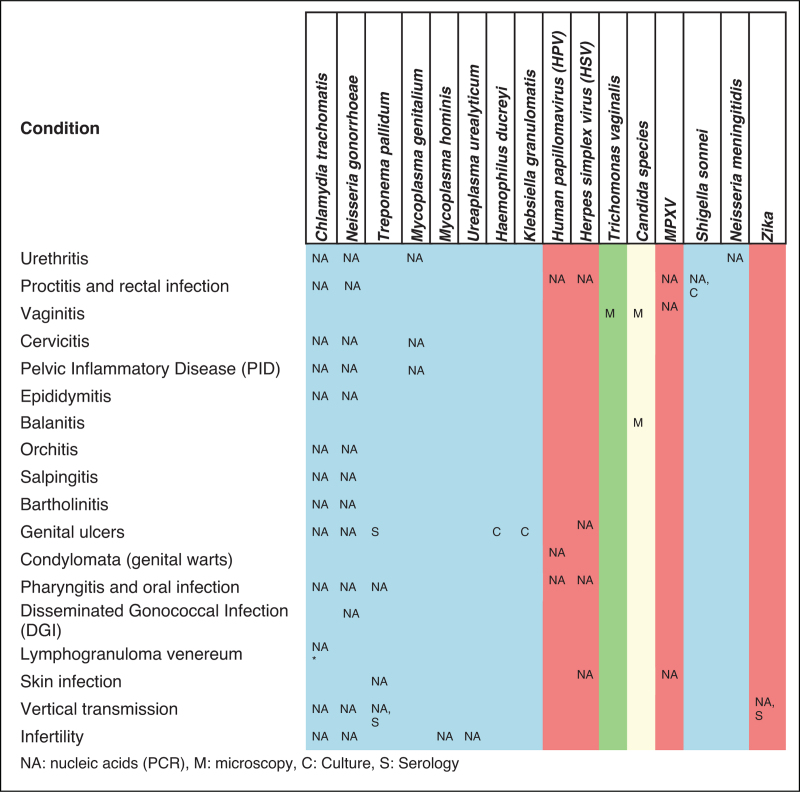
Sexually transmitted infections syndromes, more common pathogens, and diagnostic approach

### Other site of infection

Proctitis is frequently linked to *C. trachomatis* and *N. gonorrhoeae*[20]. PCR plays a crucial role in these cases, particularly for asymptomatic infections or where clinical presentations overlap. Emerging pathogens like *Shigella sonnei* and Monkeypox virus have also been implicated in proctitis. PCR's ability to quickly differentiate these pathogens is essential for guiding appropriate treatment strategies [21,22]. Pharyngitis, though less commonly caused by STIs, can result from *C. trachomatis*, *N. gonorrhoeae*, or *T. pallidum*. In such cases, PCR improves diagnostic accuracy, particularly when throat swab cultures may yield false negatives or when *T. pallidum* needs differentiation from other pharyngeal infections. Disseminated gonococcal infection (DGI), a severe complication of untreated *N. gonorrhoeae*, can present with systemic symptoms such as arthritis, tenosynovitis, or meningitis. PCR is particularly useful for detecting gonococcal DNA in blood or joint fluid, providing a rapid and reliable diagnostic tool for such disseminated cases, which are often challenging to diagnose via culture due to the limited number of bacteria in the blood or in the joint fluid [23,24].

### Emerging and zoonotic pathogens

Emerging and zoonotic pathogens pose new challenges for syndromic STI diagnostics. Recent outbreaks and evidence of sexual transmission have necessitated their inclusion in diagnostic panels. Zika Virus, though primarily mosquito-borne infection, can be sexually transmitted [25,26]. Its severe complications, particularly in pregnancy, highlight the need for rapid diagnosis. PCR assays for Zika virus have been integrated into syndromic panels for suspected sexual transmission [4]. Monkeypox virus (MPXV), initially a zoonotic infection, has demonstrated human-to-human transmission, particularly through close contact, including sexual activity. During the 2022 global outbreak, MPXV was frequently associated with genital lesions resembling syphilis or herpes [27]. PCR-based panels now often include MPXV to identify co-infections during outbreaks. Cases of MPXV-related proctitis, with or without visible lesions, have also been reported [28^▪^,^29^]. *N. meningitidis*, while traditionally linked to meningitis and septicemia, is now recognized as a cause of urogenital and anorectal infections. Its role in urethritis outbreaks emphasizes the need for its inclusion in diagnostic panels, particularly when symptoms mimic common STIs [30^▪▪^,^31^]. *Shigella sonnei*, typically an enteric pathogen, has emerged as an STI, particularly in MSM [32]. It presents with gastrointestinal and anorectal symptoms that overlap with those of STIs. Including *S. sonnei* in diagnostic panels ensures accurate differentiation and appropriate treatment. A number of intestinal pathogens are also more common in MSMs such as *Entamoeba histolytica* and *Giardia lamblia*, both nowadays easy to detect with PCR panels.

### Resistance detection and virulence factors

The identification of drug resistance mechanisms and virulence factors is essential for managing STIs, as these factors influence infection outcome and treatment decisions. Advances in PCR-based diagnostics enable rapid detection of resistance markers and virulence genes, improving clinical outcomes. *M. genitalium* poses significant treatment challenges due to increasing resistance to first-line antibiotics, particularly macrolides like azithromycin. Macrolides resistance is often linked to mutations in the 23S rRNA gene. PCR assays can detect these mutations, enabling early adjustment of treatment. Fluoroquinolone resistance, associated with mutations in the *parC* gene, is also rising and can be similarly detected by PCR [33,34^▪^]. However, even in the absence of *parC* mutations, resistance may still occur due to mutations in ParE, GyrA or GyrB encoding genes. *N. gonorrhoeae* has developed resistance to nearly all antibiotic classes, including penicillin, tetracyclines, and fluoroquinolones. The emergence of strains with reduced susceptibility to third-generation cephalosporins, such as ceftriaxone, is particularly concerning. Molecular diagnostics focus on detecting resistance-associated mutations in key genes, such as *penA* for cephalosporin resistance, *gyrA* for fluoroquinolone resistance, and 23S rRNA for azithromycin resistance [35,36]. Lymphogranuloma venereum (LGV), an invasive form of chlamydial infection caused by *C. trachomatis* serovars L1, L2, and L3, has re-emerged, particularly in MSM. It often presents as proctitis and requires prolonged antibiotic treatment, typically with doxycycline. PCR-based assays targeting the *ompA* gene differentiate LGV from non-LGV strains, guiding appropriate therapy [37]. 23S rRNA macrolide resistance mutations in *Treponema pallidum* have also emerged rapidly in communities using macrolides as oral alternatives to benzathine penicillin.

### Commercial versus laboratory developed PCR-based assays

Commercial multiplex PCR assays, such as Roche Cobas 4800, Hologic Panther, and Cepheid Xpert, are widely used for syndromic STI diagnostics. These platforms offer comprehensive, all-in-one solutions for detecting a broad range of STIs. Point-of-care (POC) molecular diagnostics, including portable PCR systems, are increasingly utilized in clinical settings, enabling rapid, on-site pathogen detection. Some commercial assays, like the Allplex system, extend coverage to less common pathogens but require multiple steps, such as separate nucleic acid extraction and amplification. Additionally, commercial assays now often include AMR markers for *M. genitalium* and *N. gonorrhoeae*, addressing rising resistance to standard antibiotics (Table 3).

**Table 3:**
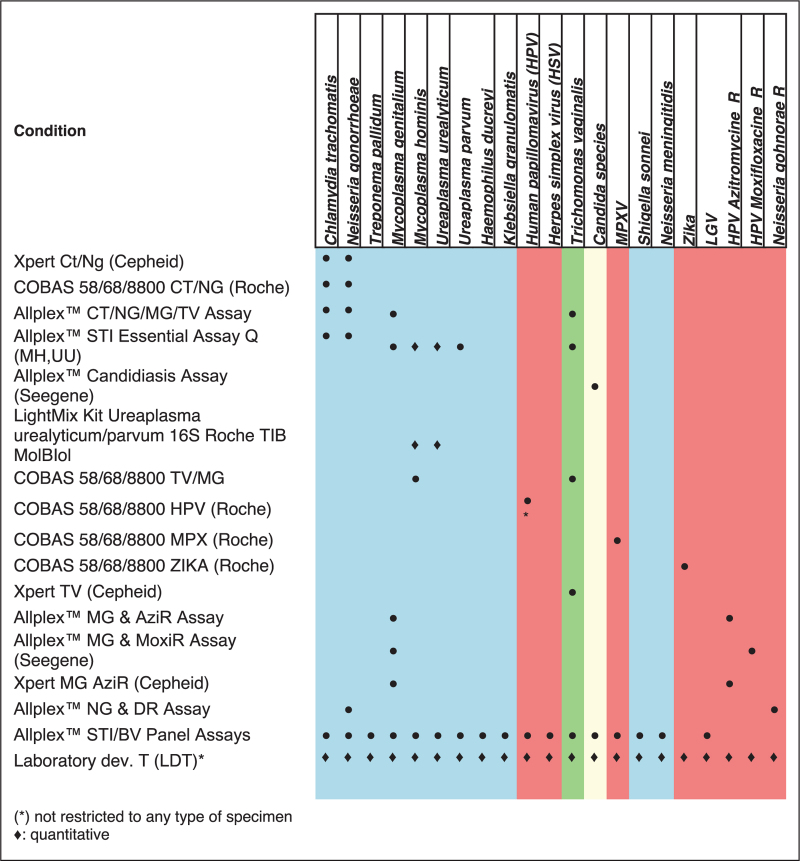
Nucleic acid base technologies for the diagnosis of sexually transmitted infection (nonexhaustive list)

Laboratory-developed tests (LDTs) complement commercial systems by offering flexibility and enhanced diagnostic precision. Unlike fixed commercial panels, LDTs enable customizable, sequential testing tailored to specific clinical needs. They can provide species-specific and quantitative detection, crucial for distinguishing colonization from infection, especially in *Mycoplasma* and *Ureaplasma* species. This allows for nuanced interpretation and better-informed treatment decisions. LDTs are particularly useful for persistent urogenital symptoms when initial tests for common pathogens are negative. Their adaptability also supports rapid development and validation of assays for emerging pathogens, such as during MPXV outbreaks, ensuring timely and precise diagnostics.

## CONCLUSION

PCR-based diagnostics have greatly improved the accuracy and speed of detecting multiple STIs. The inclusion of emerging pathogens like MPXV in syndromic panels reflects the shifting landscape of STI diagnosis. These panels address challenges such as overlapping symptoms, co-infections, and atypical presentations, enabling more informed treatment decisions. However, keeping diagnostic platforms updated to include new or re-emerging pathogens remains a challenge. Syndromic approaches must also balance sensitivity and specificity to avoid overdiagnosis and overtreatment, which can drive antibiotic resistance. Broad commercial panels, while efficient, may include irrelevant tests in some cases, leading to unnecessary resource use or treatment. Many also lack quantitative data, critical for assessing pathogen load. LDTs complement these by offering targeted, quantitative, and sequential testing, improving diagnostic precision. However, integrating LDTs and ensuring they stay current requires continuous validation and quality control.

The future of syndromic diagnostics will likely combine molecular techniques, digital tools, and data-driven methods for greater accuracy and speed to provide diagnostic stewardship. Machine learning could enhance diagnostics by identifying patterns in complex data, while integrating AMR gene detection within panels will be key to guiding effective treatments. Enhanced diagnostic strategies that combine pathogen identification with AMR profiling will become standard, improving patient care and public health responses. Collaboration across sectors will be essential to drive these advancements.

## Acknowledgements

*We thanks all the biomedical technicians of the Laboratoratory of Molecular Diagnostic of the Institute of Microbiology participating in the PCR-based routine diagnostic of STI.*

### Financial support and sponsorship

*None.*

### Conflicts of interest

*There are no conflicts of interest.*

## REFERENCES AND RECOMMENDED READING

Papers of particular interest, published within the annual period of review, have been highlighted as:

▪ of special interest
▪▪ of outstanding interest
